# Cavity-free plasmonic nanolasing enabled by dispersionless stopped light

**DOI:** 10.1038/ncomms5972

**Published:** 2014-09-17

**Authors:** Tim Pickering, Joachim M. Hamm, A. Freddie Page, Sebastian Wuestner, Ortwin Hess

**Affiliations:** 1 The Blackett Laboratory, Department of Physics, Imperial College London, South Kensington Campus, Prince Consort Road, London SW7 2AZ, UK

## Abstract

When light is brought to a standstill, its interaction with gain media increases dramatically due to a singularity in the density of optical states. Concurrently, stopped light engenders an inherent and cavity-free feedback mechanism, similar in effect to the feedback that has been demonstrated and exploited in large-scale disordered media and random lasers. Here we study the spatial, temporal and spectral signatures of lasing in planar gain-enhanced nanoplasmonic structures at near-infrared frequencies and show that the stopped-light feedback mechanism allows for nanolasing without a cavity. We reveal that in the absence of cavity-induced feedback, the subwavelength lasing mode forms dynamically as a phase-locked superposition of quasi dispersion-free waveguide modes. This mechanism proves remarkably robust against interface roughness and offers a new route towards nanolasing, the experimental realization of ultra-thin surface emitting lasers, and cavity-free active quantum plasmonics.

## Abstract (web summary)

The stopped light principle engenders an inherent feedback mechanism for light. Pickering *et al*. study the implications of this local feedback in gain-enhanced plasmonic nanostructures and find the natural emergence of ultrafast photonic and plasmonic cavity-free lasing on the nanoscale.

## Graphical abstract

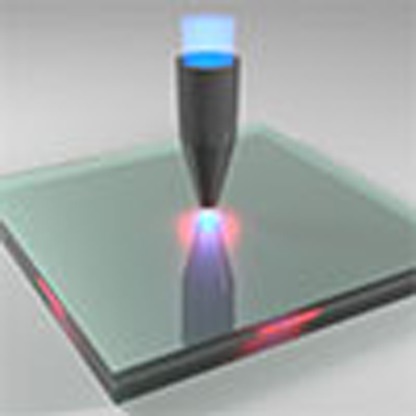

The lasing principle, one of the key discoveries of quantum-electrodynamics, has had tremendous impact on optics research and technology. In a development not too dissimilar to the revolution in transistor-based digital electronics, lasers have undergone a continuous miniaturization that culminated in the recent experimental demonstration of subwavelength nanolasing^1^,^2^,^3^,^4^,^5^,^6^. This evolution was achieved through innovative design concepts as well as advancements in fabrication techniques, which together led to breakthroughs in the two key ingredients that make a laser: light amplification and feedback. Low-dimensional semiconductor gain materials such as quantum wells and dots proved as important in shrinking lasers as did microcavity resonator designs^7^, for example, in the form of microdisks^8^, micropillars^9^, defect-mode photonic crystals^10^,^11^ and photonic nanowires. Advancing beyond traditional cavity concepts, recent nanolasers employ plasmonic resonances for feedback^12^,^13^,^14^,^15^, allowing them to concentrate light into mode volumes that are no longer limited by diffraction. The use of localized surface plasmon resonances as cold-cavity modes, however, is only one route to lasing on subwavelength scales. Lasing, in fact, does not require modes predefined by geometry but merely a feedback mechanism. To illustrate this point, one may consider the situation in a random laser where feedback arises from multiple random scattering events that lead to the spontaneous formation of closed optical paths within the disordered medium^16^,^17^. Here we show that cavity-free lasing is not restricted to micrometre sizes in disordered media (using random feedback) but can even be realized on subwavelength scales using stopped light (SL) to provide local feedback.

The fundamental principle behind SL lasing is to trap and amplify photons in space just at the point of their emission^18^. For this, a slow light regime needs to be entered, which could be achieved, for example, in photonic crystal waveguides via coherent backscattering^19^,^20^, through symmetry breaking in axially uniform waveguides^21^,^22^, or in metamaterials^23^ through the plasmonic analogue of electromagnetically induced transparency^24^. Planar plasmonic heterostructures present an even simpler design in which the stopping of light arises from the balancing of opposing energy flows in layers of materials with positive (dielectric) and negative (metal) permittivities. This design approach offers precise control over the group velocity without the need of structuring along the direction of propagation^25^,^26^,^27^. At the SL point, the overall energy flow effectively cancels, forming a characteristic closed-loop vortex^28^ (Fig. 1a), which results in a strong enhancement of the local density of optical states (LDOS) that is only limited by dissipative loss. Combined with gain, this SL feedback mechanism can then lead to coherent amplification of the trapped photons via stimulated emission processes.

Above the threshold, where the gain exceeds the losses, a localized SL lasing mode forms, whose mode volume is only limited by the waveguide dispersion. To enable subwavelength localization of the lasing mode, a broad spectrum of *k*-modes (*k* denoting the wavevector) needs to be supported by the gain (Fig. 1c). This can be achieved by introducing two (or more) SL points between which a monotonous behaviour of the dispersion is enforced. Adjusting the layer thicknesses and choice of materials allows us to simultaneously maximize the modal gain and minimize the dispersion (Fig. 1c). Although being conceptually simple, this principle can be applied universally to render the dispersion of the SL laser flat by structural optimization using, for example, genetic algorithms (see Methods).

In the following, we first determine the dispersion properties of an SL lasing system that is based on a leaky-mode metal-dielectric waveguide design. Next, we study the transient SL lasing behaviour when a finite gain section is introduced, observing ultra-fast relaxation dynamics and high-*β* characteristics that are caused by a bundling of passive *k*-modes into a single SL lasing mode. For the same structure, we offer numerical evidence that the SL points are retained up to a certain threshold value of the surface roughness, beyond which light increasingly concentrates in plasmonic hotspots on a deep subwavelength scale. Finally, we identify the mode-locking mechanism that leads to the self-localization of light on subwavelength scales and verify its applicability to both the SL nanolaser and an alternative SL spasing design.

## Results

### Dispersionless SL in metal-dielectric waveguides

We demonstrate SL nanolasing at telecom (near-infrared) wavelengths using the example structure of a planar metal-dielectric-metal stack with a top metal layer of thickness *t*=500 nm and a waveguide core of height *h*=290 nm on top of a thick metal substrate (Fig. 2a). This is a principle study, but for specificity, the dielectric permittivities of the core and cladding materials are chosen to be characteristic of III–V semiconductors (such as InGaAsP) and transparent conductive oxides (indium tin oxide) (see Methods), respectively. This particular choice can, for example, for a direct comparison with experiment, be adapted, particularly to take advantage of recent advances in improving the properties of plasmonic materials^29^. The choice of structure and materials gives rise to a (weakly) leaky TM_2_ mode in the dispersion diagram (Fig. 2b) which, by design, features two SL points that lie close in frequency: SL_1_ at *ω*_1_/2*π*=193.8 THz (*λ*_1_=1,546.9 nm) and *k*_1_=0, and SL_2_ at *ω*_2_/2*π*=193.78 THz (*λ*_2_=1,547.06 nm) and *k*_2_=1.42 μm^−1^. With both SL points situated inside the light cone, we expect the SL lasing mode to couple to free-space radiation perpendicular to the plane. The emission of light can then be controlled within limits by adjusting the thickness of the top metal cladding and amounts, for the given geometry, to around 3%–4% of the total energy lost per cycle. We initially focus on this photonic (that is, leaky) design, but will demonstrate later that it is equally possible to realize plasmonic (that is, spasing^12^) designs, where multiple SL points fall onto a bound plasmonic mode.

Having established the dispersionless character of the SL mode using a passive mode theory (Fig. 2b), we proceed to find the threshold conditions for SL lasing by performing a small-signal gain analysis. We assume that the gain medium fills the dielectric core of the structure, except for a 10-nm buffer next to the metal, which is introduced to account for gain quenching in close vicinity to the metal. The analytic dispersion equations are solved for varying levels of inversion (see Supplementary Table 1 for the gain material parameters). As the two SL points retain their positions and effectively pin down the dispersion between them, no significant change of the modal dispersion is observed with increasing inversion (Fig. 2c). In contrast, the modal loss shows a strong dependence on the inversion and changes sign at Δ*N*_th_≈0.13 (Fig. 2c, bottom). At this point, the mode becomes undamped and, owing to the flatness of the band, experiences an almost uniform level of gain over a broad range of *k*-values (up to *k*≈5 μm^−1^ where the dispersion *ω*(*k*) ultimately loses the gain support). As the calculated loss rate includes both dissipative and radiative (leakage through the cladding layer) contributions, we can associate Δ*N*_th_ with the threshold inversion required for lasing.

### Dynamics of cavity-free SL lasing

To gain insight into the transient dynamics and characteristics of SL lasing on subwavelength scales, we perform first-principles time-domain simulations, taking into account the nonlinearity of the gain material as well as the spatio-temporal interplay between light-field and inversion (Fig. 3). The gain material, described by four-level Maxwell-Bloch equations^14^ with parameters that match those used in the semi-analytical mode calculations above, is initially confined to a 400-nm-wide section, approximately four times smaller than the vacuum wavelength at the SL points. By design, the gain section can be pumped optically using a propagating waveguide mode at a higher frequency (Supplementary Fig. 1 and Supplementary Table 2). At a pump level well above threshold, the transient field and inversion dynamics (Fig. 3a) display ultrafast relaxation oscillations with a period of around 10 ps. The steady-state inversion settles at ~0.5, a value significantly higher than the threshold inversion predicted by our semi-analytical calculations (Fig. 2b). This increase in the inversion is the result of a reduced confinement of the fields to the now finite gain section (confinement factor Γ≈0.31, as defined in the Supplementary Discussion), additional losses along the waveguide due to group velocity dispersion and spatial hole burning effects caused by the inhomogeneity of the localized SL-mode profile (Fig. 3b). The laser output energy follows a familiar *S*-shaped input–output curve (Fig. 3c) with a threshold pump rate *r*_p_≈1.43 ps^−1^. Spectral narrowing of the emission line (insets of Fig. 3c) above the lasing threshold is due to the build-up of phase coherence in the SL mode, while below threshold we observe amplified spontaneous emission with a spectrum that matches the spectral density of states of the TM_2_ mode at *k*=0.

**Figure 1:**
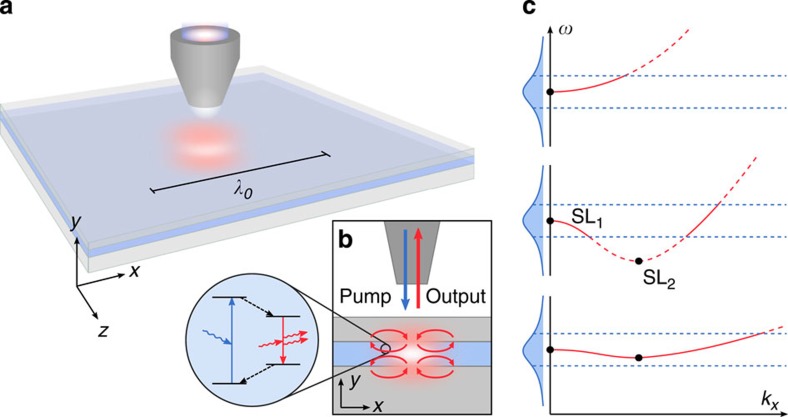
Principle of a cavity-free SL laser supporting subwavelength light localization. (**a**) The core layer of the metal-dielectric (SL) multilayer structure is filled with gain material (blue). (**b**) Spatially selective excitation of the homogeneous gain layer using a near-field tip leads to the formation of a subwavelength spot of inverted gain, in which the stimulated emission processes take place (inset). Photons are trapped locally in a closed-loop energy vortex (red curved arrows), enabled by an SL point, SL_1_, at (*ω*_1_, *k*_1_) that aligns with the peak gain. (**c**) A second SL point, SL_2_, at (*ω*_2_, *k*_2_) enforces a monotonous behaviour of the dispersion over a range of wavevectors with an average slope of (*ω*_2_−*ω*_1_)/(*k*_2_−*k*_1_). Bringing the frequencies of the SL points close together while maintaining a large distance in *k*-space flattens the dispersion to within the bandwidth of the gain (blue), allowing for the formation of highly localized, SL wave packets during lasing operation.

**Figure 2:**
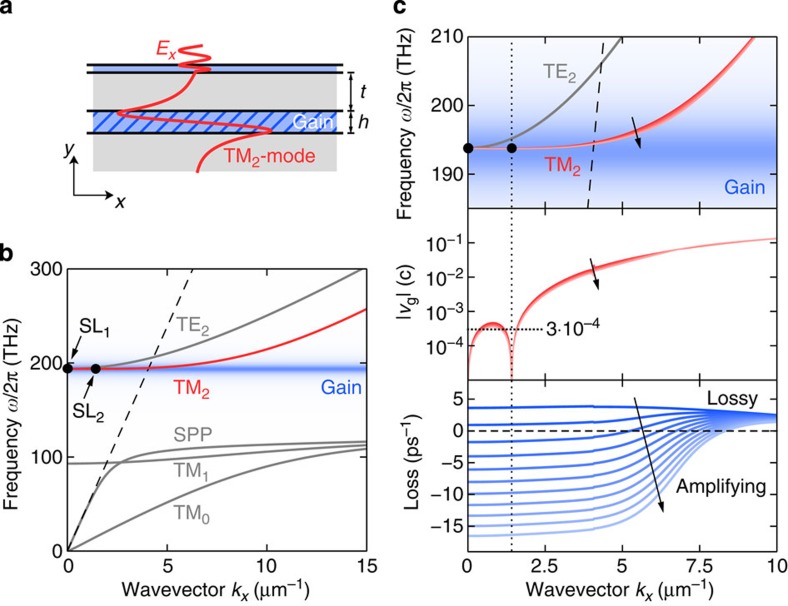
Dispersion and small-signal gain analysis of the planar SL structure. (**a**) The metal-dielectric structure with gain section height *h*=290 nm and top metal layer thickness *t*=500 nm is designed to support a weakly leaky TM_2_ mode at a wavelength of 1,547 nm. The layer thickness *t* can be tuned to control the emission of radiation. (**b**) Passive case: the (complex frequency) dispersion of the TM_2_ mode exhibits two SL points (marked with black circles) within the light cone (dashed line) that are lined up with the gain spectrum (in blue). A thin dielectric cover layer pushes the SPP branch, which would otherwise intersect the gain spectrum at higher wavevectors resulting in mode competition, to lower frequencies. (**c**) When gain is introduced into the core the modal dispersion, group velocity and modal loss (top to bottom) will change depending on the gain coefficient ranging from 0 cm^−1^ (no inversion; dark colour) to 4,180 cm^−1^ (maximum inversion; light colour) as indicated by the arrows. The analysis highlights the robustness of the SL points, leading to a flat dispersion of average velocity ~3 × 10^−4^*c* that is practically not affected by the level of inversion. The dashed line in the top panel represents the light line.

Owing to a vanishing group velocity of the lasing TM_2_ mode, the LDOS is strongly enhanced around the frequency of the SL points (Supplementary Fig. 2). The TE_2_ mode, which shares the SL point at *k*=0 with the TM_2_ mode (Fig. 1), offers a competing channel for spontaneous emission but its LDOS reaches only about one-tenth of the value of the TM_2_ mode due to its stronger dispersion. Consequently, 90% of the spontaneous emission are channelled into the dispersionless TM-polarized lasing mode, which is confirmed by the spontaneous emission factor obtained from the threshold curve, *β*=*γ*_c_/(*γ*_c_+Γ*r*_p_)≈0.9 (ref. 30) (total loss rate *γ*_c_≈3.71 ps^−1^; Supplementary Fig. 3). In contrast to microcavity lasers, where spontaneous emission channels can be suppressed via cavity design, the concentration of the spontaneous emission into the localized SL lasing mode is a result of the large density of *k*-modes that fall within the bandwidth of the gain. The observed high-*β* characteristics and picosecond relaxation oscillations of cavity-free SL lasing can potentially allow for the design of thresholdless plasmonic laser diodes that can be modulated with THz speeds.

### Resilience to surface roughness

It has been shown that even small structural imperfections can have a significant impact on the propagation characteristics near SL points in slow light devices^20^. The increase of scattering at interfaces not only imposes limitations on the achievable group index but can also result in Anderson localization of light, as has been demonstrated for photonic crystal waveguides^31^. In metal dielectric heterostructures, however, SL arises from negative energy flow inside the metallic cladding and not from backscattering at geometric features^32^. It is therefore important to determine the sensitivity of the SL feedback mechanism to structural imperfections that are likely to be present in any experimental realization.

We study the influence of surface roughness on SL lasing by applying a normally distributed variation of surface roughness (height in *y* position) at the interfaces between the core and the cladding layers along the length of the waveguide with a fixed step size of 10 nm in *x* direction. The autocorrelation of the resulting roughness profiles follows a Gaussian distribution with an associated correlation length of ~8.5 nm. SL lasing is investigated in structures with roughness heights of root-mean-square (r.m.s.) values between 0 and 3 nm. For each r.m.s. height, the total loss rate is averaged over ten lasing configurations (shown in Fig. 4).

We find that SL lasing remains resilient to surface roughness with almost no variation in the average loss rate below r.m.s. values <1.5 nm. Above this r.m.s. value, however, both the average loss rate and s.d. steadily rise. In addition, for r.m.s. heights above 2.5 nm, an increasing number of simulations do not show SL lasing; for example, ~50% of the configurations at 3 nm r.m.s. surface roughness do not lase. The higher losses arise primarily from the scattering of the modal fields at the rough interfaces and an increased absorption of energy at hotspots, surface irregularities over which the SL lasing mode localizes as demonstrated in the mode profiles for different levels of r.m.s. roughness (insets of Fig. 4). This remarkable resilience of SL points to surface roughness can also be observed in the passive metal-dielectric heterostructure (Supplementary Fig. 4) by measuring the rise of the average energy velocity with the interfacial roughness.

## Discussion

Our analysis of the lasing dynamics shows that the lasing mode localizes in-plane to the subwavelength gain region even though there is no cavity that would enforce this behaviour. To explain the mechanism that governs the formation of the subwavelength modes, we consider two distinct cases: SL nanolasing in the previously introduced leaky-mode design and SL spasing using an alternative plasmonic-mode design that allows for plasmon amplification.

**Figure 3:**
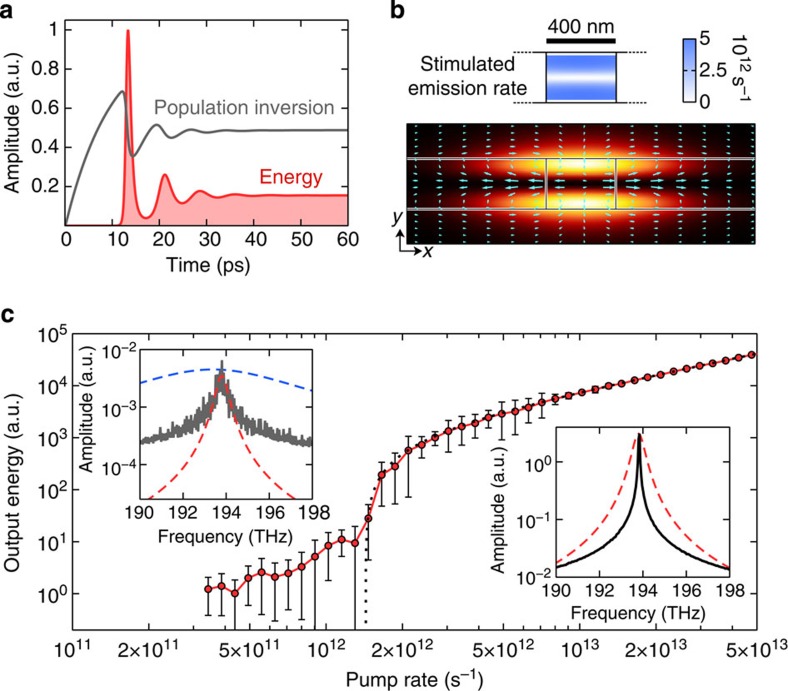
Nonlinear lasing dynamics and laser threshold behaviour. (**a**) Characteristic single-mode relaxation oscillations with a period of around 10 ps are observed in the temporal evolution of the population inversion and the field energy for a metal-dielectric SL structure with a 400-nm wide gain section. (**b**) The intensity profile of the steady-state lasing mode has TM_2_ character and strongly localizes over the gain section (highlighted by the vertical lines). The Poynting vectors (cyan) form a closed-loop energy vortex that provides the feedback mechanism for the SL laser. Stimulated emission into the SL lasing mode takes place in the upper and lower parts of the gain section (top) causing strong spatial hole burning in the inversion. (**c**) The laser output energy shows an *S*-shaped input–output curve with a threshold pump rate of *r*_p_=1.43 ps^−1^. A comparison of the amplified spontaneous emission (ASE) spectrum below threshold (left inset) and the lasing spectrum (right inset) indicates spectral narrowing due to the build-up of phase coherence in the SL mode. Close to its maximum value, the ASE spectrum closely follows the spectral density of states of the TM_2_ mode at *k*=0 (red dashed line, normalized to the ASE and lasing peak values, respectively) and is much narrower than the gain spectrum (blue dashed line). The output energy and its error in **c** were determined during steady-state emission as the average value and s.d. of the cycle-averaged electromagnetic energy in a fixed time window (35 ps above and 200 ps below threshold).

**Figure 4:**
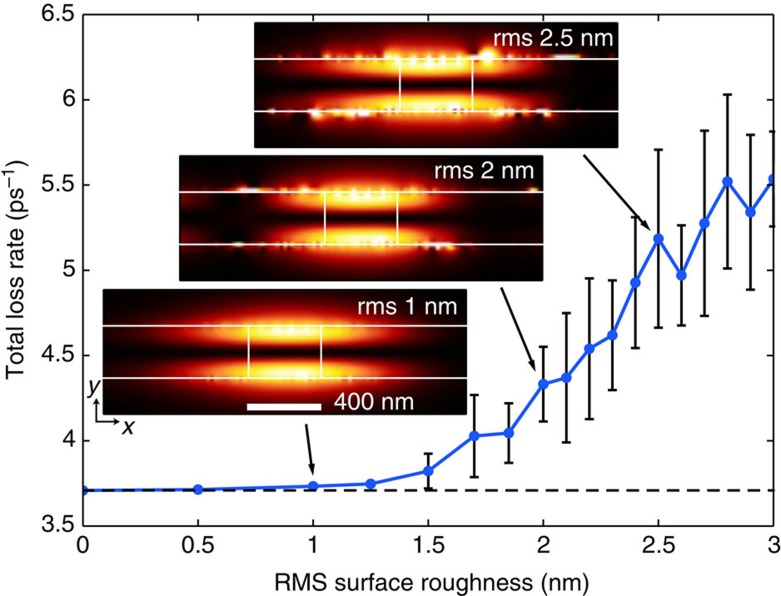
Impact of surface roughness on SL lasing. For small values of surface roughness the total loss rate of the lasing mode is weakly dependent on the r.m.s. surface roughness. Above r.m.s. values of 1.5 nm, a linear increase is observed. Surface roughness leads to the appearance of local hotspots in the time-averaged intensity profile during steady-state lasing, shown here for increasing levels of surface roughness: 1, 2 and 2.5 nm from bottom to top. The total loss rate and its error were calculated as the average loss rate and s.d. from ten lasing configurations for each r.m.s. surface roughness value.

**Figure 5:**
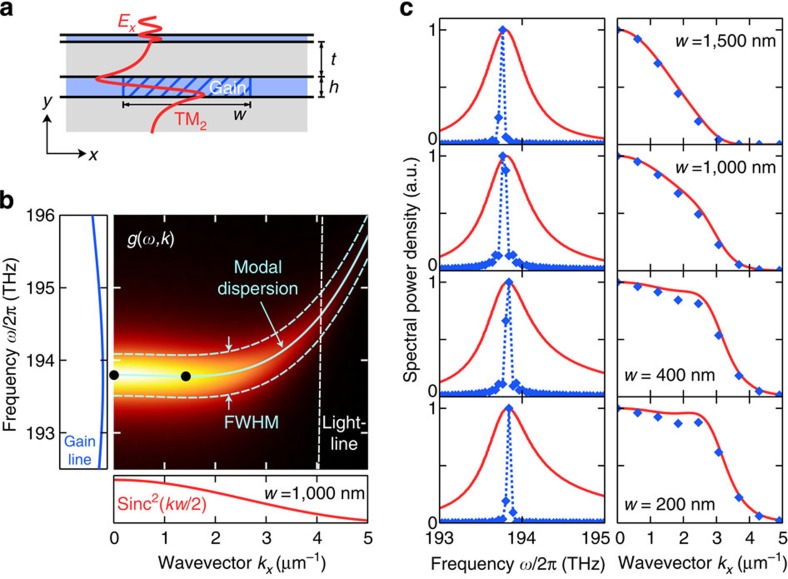
Frequency selection and mode formation in an SL nanolaser. (**a**) *E*_*x*_ field profile of the TM_2_ mode along the *y* direction overlaid on the spatially confined section of inverted gain (blue hatched region). (**b**) Semi-analytical calculation of the coupling strength *g*(*ω*, *k*) (heat map) as the product of the gain spectrum (*ω*−*ω*_0_) (blue line), the density of optical states *D*(*ω*, *k*) of the TM_2_ mode (cyan line) and the sinc^2^(*kw*/2) spectrum of the rectangular gain section (red line). (**c**) Prediction of the lasing frequency (left column) and the mode profile (right column) for successively smaller gain sections showing the effect of an increasing localization of the SL mode. The results confirm that the SL laser selects a lasing frequency *ω*_SLL_ (blue diamonds) at which *G*(*ω*)=∫ d*k*g(*ω*, *k*) (red line) peaks. The mode shape (right column) is then determined by *g*(*ω*_SLL_, *k*) (red line), which is in excellent agreement with the numerically extracted *k*-spectrum of the mode profile (blue diamonds).

In contrast to conventional single-mode lasing, where the lasing frequency and mode profile are determined by the cavity, the SL lasing mode is characterized by a closed-loop energy vortex (Fig. 3c) and forms dynamically by phase-locking the continuum of *k*-modes in the vicinity of the SL points. During the transient phase, the lasing mode acquires a spectral composition that maximizes the effective gain *G*(*ω*)∝∫d*k* (*ω*−*ω*_0_) · *D*(*ω*, *k*) · sinc^2^(*kw*/2) =∫d*k g*(*ω*, *k*) by balancing the spatial confinement of the lasing mode to the gain section against its spectral overlap with the gain line (*ω*−*ω*_0_). The density of states *D*(*ω*, *k*)∝*γ*(*k*)/[(*ω*(*k*)−*ω*)^2^+*γ*^2^(*k*)] of the (TM_2_) waveguide mode plays a critical role in this process as it links and controls the spatial and spectral overlap. The close agreement of the analytically predicted frequencies *ω*_SLL_ and *k*-spectra, *g*(*ω*_SLL_, *k*), with those retrieved from full spatio-temporal simulations (Fig. 5c) confirms this principle: the frequency *ω*_SLL_ of the SL lasing mode is selected by maximization of *G*(*ω*), and its mode shape is determined by *g*(*ω*_SLL_, *k*). The results further suggest that there is no fundamental limit for the spatial compression of the mode, although a higher localization requires an increasingly flat dispersion to maintain a high spectral overlap.

**Figure 6:**
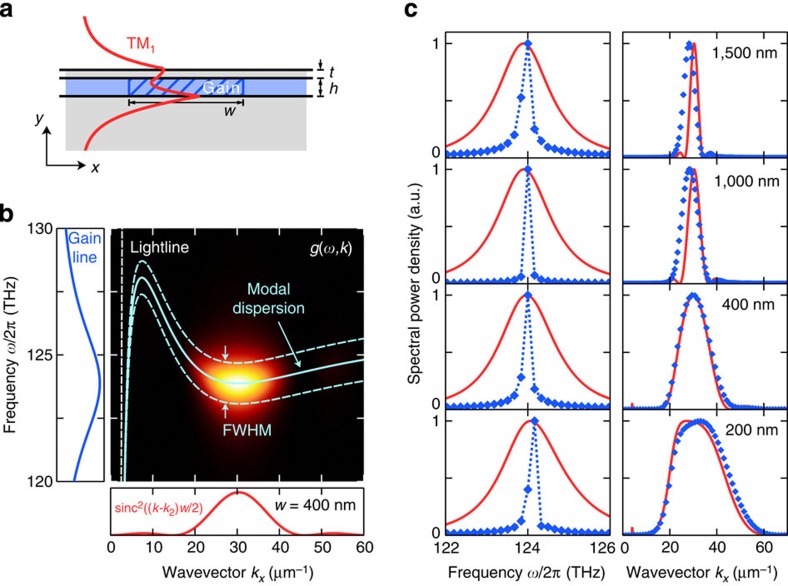
Frequency selection and mode formation in an SL spaser. (**a**) *E*_*x*_ field profile of the TM_1_ mode along the *y* direction overlaid on the spatially confined section of inverted gain (blue hatched region) in a metal-dielectric stack structure with waveguide core height *h*=110 nm and top metal layer thickness *t*=50 nm. (**b**) Semi-analytical calculation of the coupling strength *g*(*ω*, *k*) (heat map) for the plasmonic TM_1_ mode. The peak of the sinc^2^ spectrum of the rectangular gain section is shifted to the second SL point with *k*=*k*_2_, because the gain line is maximum at the frequency of this SL point. (**c**) Prediction of the lasing frequency (left column) and the mode profile (right column) for successively smaller gain sections. The SL laser selects a lasing frequency *ω*_SLL_ (blue diamonds) at which *G*(*ω*)=∫d*k*g(*ω*, *k*) (red line) peaks, leading to the *k*-spectrum of the mode profile (blue diamonds) to coincide with *g*(*ω*_SLL_, *k*) (red line).

With bound plasmonic modes one can exploit a wider range of wavevectors and achieve considerably smaller effective mode volumes. We optimized the waveguide dispersion (Fig. 6b and Supplementary Fig. 5) to exhibit two SL points in the plasmonic TM_1_ mode at *ω*_1_/2*π*=128.14 THz (*λ*_1_≈2,339.6 nm) at *k*_1_=7.41 μm^−1^ and *ω*_2_/2*π*=123.89 THz (*λ*_2_≈2,419.7 nm) at *k*_2_=30.34 μm^−1^. There is no outcoupling to propagating free-space waves and the modal loss is approximately three times larger than the total loss of the TM_2_ waveguide mode due to the higher field localization. Figure 6b shows the predicted coupling strength *g*(*ω*,*k*) for a plasmonic SL lasing structure. We choose to tune the emission line of the gain medium to the frequency of the second SL point *ω*_2_ (Supplementary Table 3) and consequently shift the peak of the the sinc^2^-function to *k*_2_. The broad spectrum of supported wavevectors allows for localization of the SL lasing mode down to gain sections with a width of *w*=200 nm. For these small gain sections, the maximum of the field profile aligns over the gain, effectively locking the amplitude and phase of the complementary modes with positive and negative phase velocities in space. As the spectrum of wavevectors is centred at a non-zero wavevector *k*_2_, the lasing mode becomes spatially modulated in propagation direction with nodes every *π*/*k*_2_. For wider gain sections, the positions of the nodal planes can move in time depending on the contributions to the lasing mode from the counterpropagating waveguide modes. This behaviour is a direct consequence of the cavity-free property of SL lasing, where forward and backward modes become uncoupled due to the absence of backreflections at geometric boundaries (compare with, for example, Fabry–Perot lasers), and will be studied in more detail in a future publication.

In both cases, the SL lasing/spasing mode emerges from the mode locking of passive modes via the gain-coupling factor *g*(*ω*,*k*). Excellent agreement is observed between the semi-analytical analysis and the numerically extracted spectra for both the lasing frequency and the wavevector spectrum over a range of gain section widths (Fig. 6c). Indeed, the results confirm that SL lasing is enabled by a universal mechanism of SL-assisted mode formation that optimizes the net gain in these structures without being limited by diffraction.

With the planar structure being isotropic in two dimensions, the generalization to three dimensions is straightforward. By introducing a small cylinder of gain material (or, for example, a quantum dot) into the waveguide core layer or by spatially selective excitation of a homogeneous gain layer, one can conceivably construct a ‘photonic-dot’ laser that, evanescently pumped by a near-field tip, would receive feedback by formation of a three-dimensional SL vortex. In the vicinity of the metal layer, we observe very high gain coupling factors due to the combined action of a vertical plasmonic confinement and the in-plane stopping of light, which together have an impact on the local density of states. The presented metal-dielectric SL structures are therefore predestined for achieving strong coupling in plasmonic structures, as recently predicted theoretically^33^. SL lasing may thus open the door to cavity-free nanolasing, ultra-thin lasing surfaces and cavity-free quantum electrodynamics. Through a flexible (multi-point) localization of lossless or amplified surface plasmon polaritons, the SL scheme also provides an entry point to quantum gain in quantum plasmonics^33^ and quantum fluids of light^34^.

## Methods

### Complex-frequency dispersion analysis

The dispersion and loss curves for the transverse magnetic (transverse electric)-polarized modes of the planar waveguide were calculated semi-analytically using a modified transfer matrix method^35^, which allows for extraction of the complex-*ω* modes that remain valid solutions at the SL points. Let us consider a planar metal-dielectric stack structure of *N* layers of thickness *d*_*i*_ (*i*=1…*N*) that are stacked up in *y* direction and extend infinitely in the *x*–*z* plane. For isotropic media, the field component *?*_*z*_, orthogonal to the propagation direction *x*, can be written as


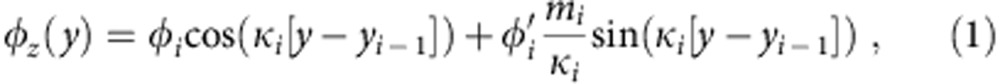


where *m*_*i*_ is the layer permittivity (permeability) for *?*_*z*_=*H*_*z*_ (*?*_*z*_=*E*_*z*_). Momentum conservation dictates that the *y* component of the wavevector 
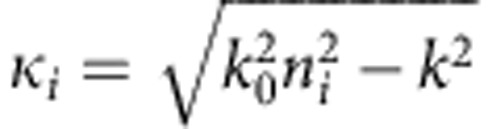
 depends on the *x* component *k* (also called propagation constant). The boundary conditions between individual layers translate into


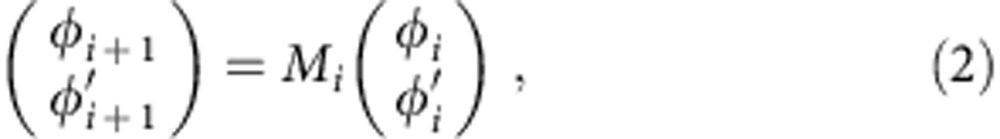


where the matrices *M*_*i*_ are given by


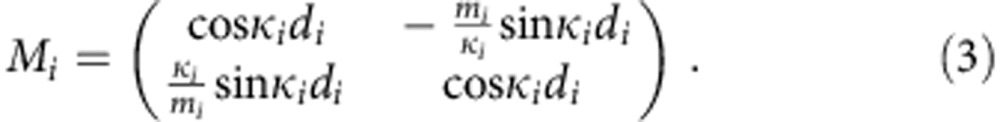


The bound mode profile leads to the initial condition 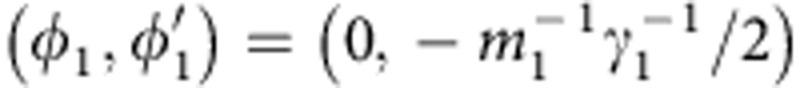 in the superstrate, while in the substrate the condition can be expressed as


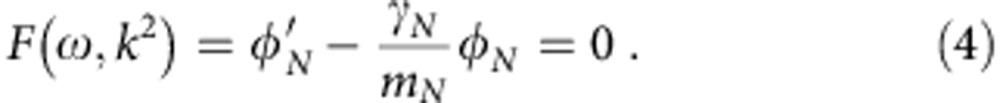


where 
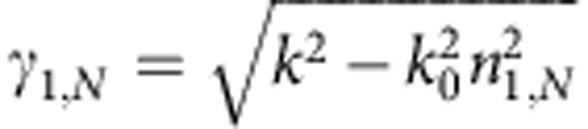
 define the decay constants in the cladding layers.

For plasmonic and active layer structures, the characteristic function *F*(*ω*, *k*^2^) is a complex-valued function. Its roots lie on a hyperplane and can be found efficiently using a combination of the argument principle and the Newton–Raphson methods.

In practice, when solving the characteristic equation one considers one of the arguments (*ω* or *k*^2^) as a real parameter and the other as complex eigenvalue. For real *ω*, the complex-*k* eigenvalue describes a mode with spatially decaying (or increasing) amplitude, a solution that resembles those obtained by cw mode injection into the waveguide. These solutions do not exist at the SL points, as the corresponding mode cannot propagate. Instead, one needs to consider complex-*ω* modes (with a fixed, real *k*). As these modes decay in time (rather than space), they do not show the typical back bending and mode splitting of the complex-*k* dispersion curves at the SL points.

The roots of the dispersion function can be classified according to their behaviour in the cladding layers. The bound modes, characterized by an exponential decay in the super- and substrate (Re[*γ*_1_], Re[*γ*_*N*_] > 0), are associated with a discrete spectrum of eigenvalues. In contrast, the radiative continuum is described by modes with a purely oscillatory behaviour (Re[*γ*_1_],Re[*γ*_*N*_]=0)^36^. The third class of modes, so-called leaky modes, show a divergent behaviour in the cladding. These modes, despite not being part of the complete basis set, are observable as resonances in the reflection spectrum. For the (weakly leaky) structure under investigation, leaky modes accurately capture the changes to the modal dispersion and the additional radiative loss caused by the coupling of the waveguide mode to the continuum of free space modes.

### Maxwell-Bloch Langevin time-domain simulations

The time-resolved simulations were performed using the finite-difference time-domain method in combination with auxiliary differential equations that describe the material dispersion of the metal cladding and the nonlinear, spatially resolved polarization response of the gain media. Using the Maxwell-Bloch formalism we modelled the gain medium as a four-level system with pump (0→3) and emission (2→1) transitions^37^ that we associate with polarization densities **P**_a_(**r**, *t*) and **P**_e_(**r**, *t*). The dynamics of the polarization densities can be mapped on a set of differential equations


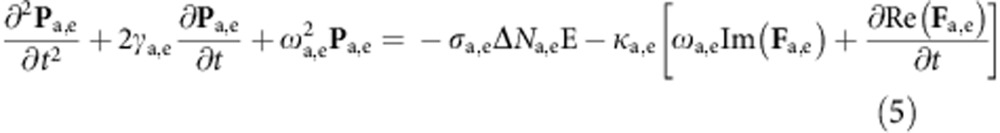


which describe the dynamics of light–matter interaction via the dipole coupling of the electric field to the electronic transitions. The resonance frequencies *ω*_*i*_ (and transition frequencies 
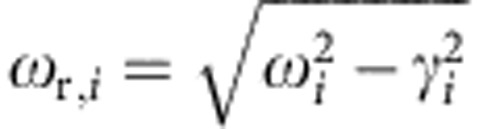
), the spectral half-widths *γ*_*i*_, the phenomenological coupling constants 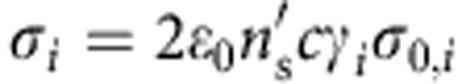 (that are connected to the emission and absorption cross-sections *σ*_0,*i*_), as well as the real part of the host refractive index 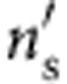 are determined by the choice of gain material.

The temporal evolution of the occupation densities *N*_0_ to *N*_3_, which in turn determine the inversions Δ*N*_e_(**r**,*t*)=*N*_2_(**r**,*t*)−*N*_1_(**r**,*t*) and 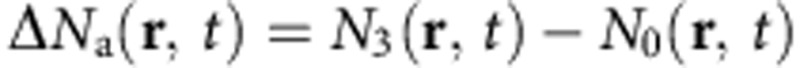 for emission and absorption, is given by the set of equations


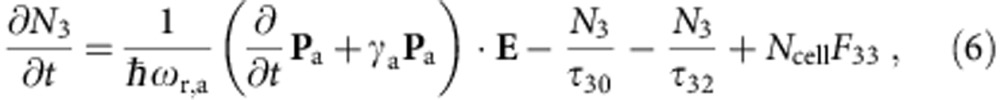



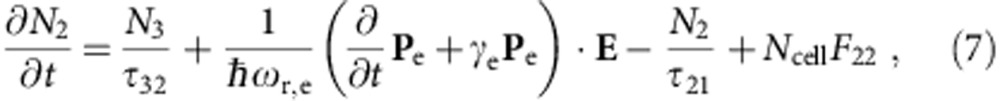



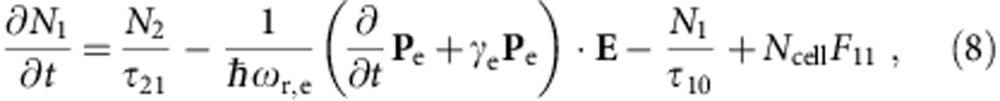



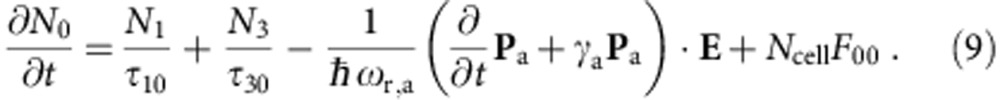


The lifetimes *τ*_*jk*_ account for non-radiative relaxation processes between the levels. When considering a spatially homogeneous and constant local pump rate 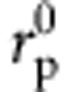, the term connected to the polarization density of the absorption transition, (*ℏω*_r,a_)^−1^(∂**P**_a_/∂*t*+Γ_a_**P**_a_)**E**, can be replaced by 
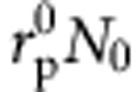
.

Dissipation-induced fluctuations of the polarizations and carrier densities are included in the Maxwell-Bloch equations via Langevin noise terms^37^. The noise terms are given by **F**_e_(**r**, *t*), **F**_a_(**r**, *t*) and *F*_00_(**r**, *t*) to *F*_33_(**r**, *t*), where *N*_cell_ is the number of four-level systems per unit volume cell of the finite-difference time-domain grid. Below the lasing threshold the noise acts as source for amplified spontaneous emission giving rise to the recorded background spectra (Fig. 3b). Above the lasing threshold, the noise acts as a seed aiding the transient build-up of the coherent lasing fields through feedback and stimulated emission processes in the gain medium.

### Structural and material parameters

The planar metal-dielectric stack structures comprises an optically thick metal substrate, a thin dielectric core layer and a second (thin) metal cladding layer. Further control over the modal dispersion is achieved by introducing a dielectric layer on top of the stack structure when necessary^26^. In our analysis, we assume a constant permittivity of *ε*_s_=11.68 for the dielectric layers, a value that is typical for III–V semiconductors (such as InGaAsP). The dispersive permittivity of the metal layers, in this case a transparent conductive oxide, is described using a Drude model. In accordance with experimental data^38^, we use *ω*_p_=3.13·10^15^ rads^−1^ for the plasma frequency of indium tin oxide. The collision rate γ is assumed to be reduced by a factor of 10 to 1.07·10^13^rads^−1^ as could be achieved by fabricating films of very high quality and cooling them to low temperatures^5^. In comparison to metals such as silver or gold, the plasma frequency for, e.g., indium tin oxide can be controlled through doping and can be shifted into the visible wavelength range to around 600 nm making this material a suitable option for SL lasing in the near-infrared.

### Genetic optimization of broadband dispersionless modes

The principle of sub-wavelength SL lasing relies on the formation of a flat band in the modal dispersion that maximizes the range of wavevectors contained within the frequency bandwidth of the gain. The flatness of the band can be achieved by introducing two or more SL points, which enforce a monotonous variation of the dispersion in between them. We employ a genetic algorithm technique to isolate structures of a given design that exhibit two SL points and the smallest possible frequency variation in a selected mode.

Every planar metal-dielectric stack structure is uniquely characterized by the number of layers, the layer thicknesses and their permittivities, forming an ordered parameter set, the so-called chromosome of the genetic algorithm. On each iteration of the algorithm, we hold a population of *N* chromosomes, which are evaluated with respect to a fitness function that returns a single numerical value representing suitability of the structure. The population is sorted to this order and a small percentage of the fittest chromosomes are passed to the next generation unchanged. The remaining places in the new population are filled by ‘tournament selection’, where each tournament is a subset of the current population whose members are chosen at random. The two fittest chromosomes from each tournament are then mutated by randomly perturbing parameters of the chromosome and optionally crossed over by swapping sections of the parameter list between the two tournament winners. The two new chromosomes are added to the next population, and the process of tournament selection is repeated until the population set is complete. The progress of each iteration can be monitored by considering the fittest chromosome in the population and the algorithm is terminated once this chromosome surpasses a threshold fitness.

For the fitness function, we first evaluate the dispersion relation of a given chromosome (that is, structure) and perform a root find for two SL points. If the calculation fails during this step, the function returns to zero, otherwise we determine the average band velocity and return its reciprocal value


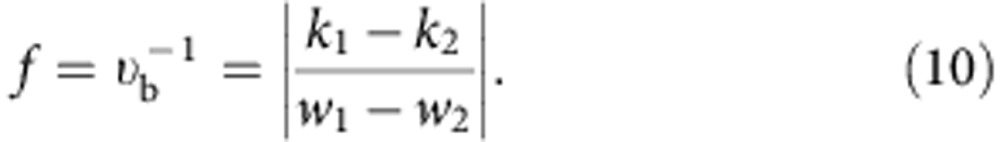


The structure with the highest value of *f* is deemed fittest. It is common to initialize the population with chromosomes representing structures already known to exhibit two SL points, which may in turn be found by optimization using a different, appropriate fitness function.

## Author contributions

T.P., J.M.H. and O.H. developed the concept and conceived the study. T.P. and J.M.H. designed and performed the complex frequency dispersion analysis. T.P., A.F.P. and S.W. performed the Maxwell-Bloch Langevin time-domain simulations. A.F.P. implemented and ran the genetic optimization. O.H. supervised the work. All authors discussed the results and wrote the manuscript.

## Additional information

**How to cite this article**: Pickering, T. *et al*. Cavity-free plasmonic nanolasing enabled by dispersionless stopped light. *Nat. Commun.* 5:4972 doi: 10.1038/ncomms5972 (2014).

## Supplementary Material

Supplementary Information Supplementary Figures 1-5, Supplementary Tables 1-3, Supplementary Discussion and Supplementary References.
