# Supplementary material for: Cavity-free plasmonic nanolasing enabled by dispersionless stopped light
Source: Nat Commun. 2014 Sep 17;5:4972. doi: 10.1038/ncomms5972 (PMC4199200; doi:10.1038/ncomms5972)
Supplement: Supplementary Information — Supplementary Figures 1-5, Supplementary Tables 1-3, Supplementary Discussion and Supplementary References. [file ncomms5972-s1.pdf]

Supplementary Figures

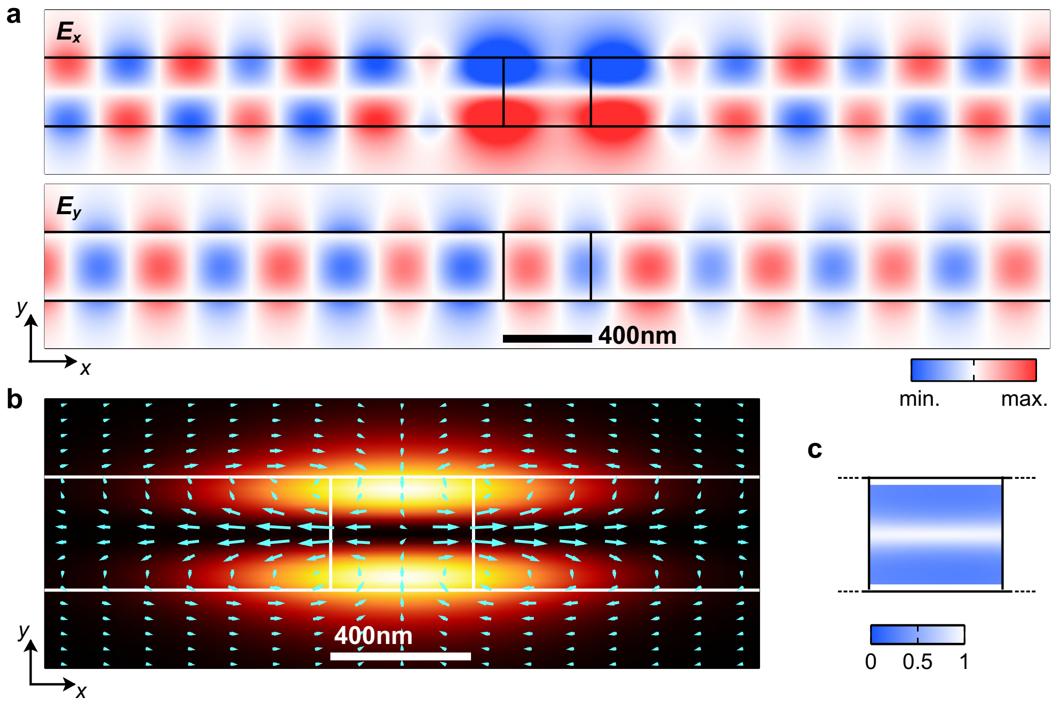

Supplementary Figure 1 | Optical pumping. (**a**) Snapshots of the electric field amplitudes E_x and E_y showing the incoming pump field (from the left) and, close to and inside the gain section, its interference with the SL lasing mode. Approximately 19% of the pump mode energy is absorbed in the 400 nm wide gain section. (**b**) The intensity profile and Poynting vectors of the steady-state lasing mode and (**c**) the associated steady-state inversion. See "Optical pumping" in the supplementary discussion for further details.

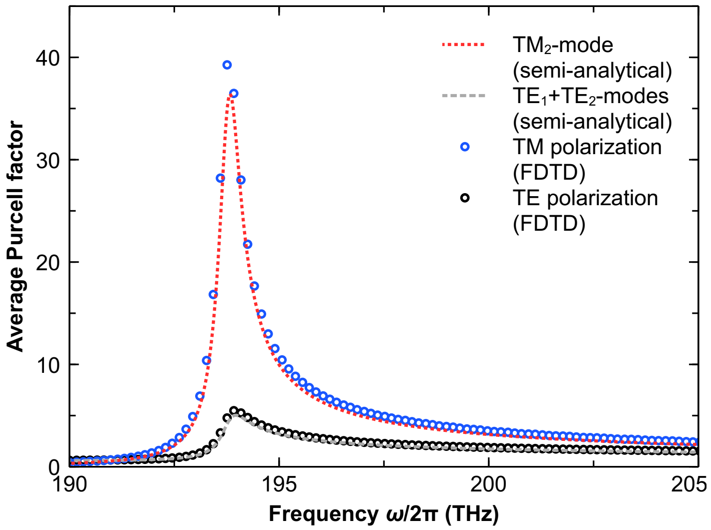

Supplementary Figure 2 | Purcell factor (enhancement of the partial local density of optical states) for emission into the TM and TE modes of the planar metal-dielectric stack. The Purcell factor is spatially averaged over all emitter positions within the waveguide core. Numerical FDTD results (blue and black circles) are compared to the semi-analytical evaluation of equation (1) (red and gray lines) using the dispersion data of the TM_2 mode and of the TE_1 and TE_2 modes, respectively. See "Density of optical states" in the supplementary discussion for further details.

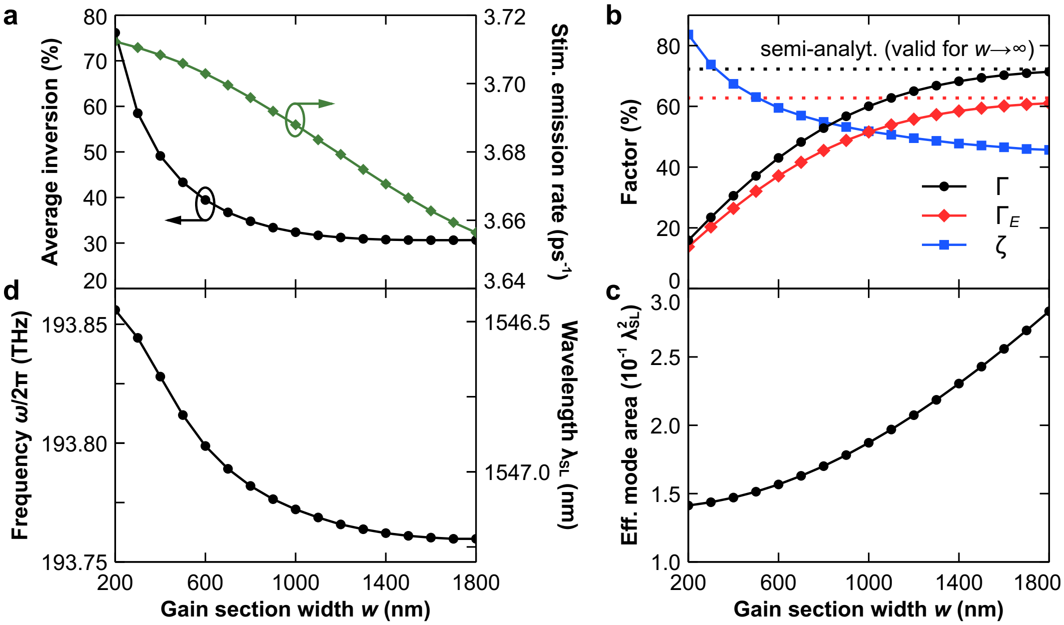

Supplementary Figure 3 | Lasing properties of the TM_2 waveguide mode. (**a**) Average inversion ΔN and stimulated emission rate R_{stim} in dependence of the gain section width w during steady-state lasing of the TM_2 mode. (**b**) Confinement factor Γ and energy confinement Γ_E of the lasing mode to the gain section in dependence of w. The factorization parameter ζ is defined in the text. Semi-analytical results for Γ and Γ_E, obtained from mode solver calculations, are represented by the dotted lines and fit well in the limit of large w. (**c**) Effective mode area calculated as A_{g}/Γ from the confinement factor in (**b**) and the gain section area $A_{\mathrm{g}} = w \cdot 270\,\mathrm{nm}$. (**d**) The spectral blue-shift of the lasing frequency with decreasing gain section width. See "Steady-state analysis for varying gain section width" in the supplementary discussion for further details.

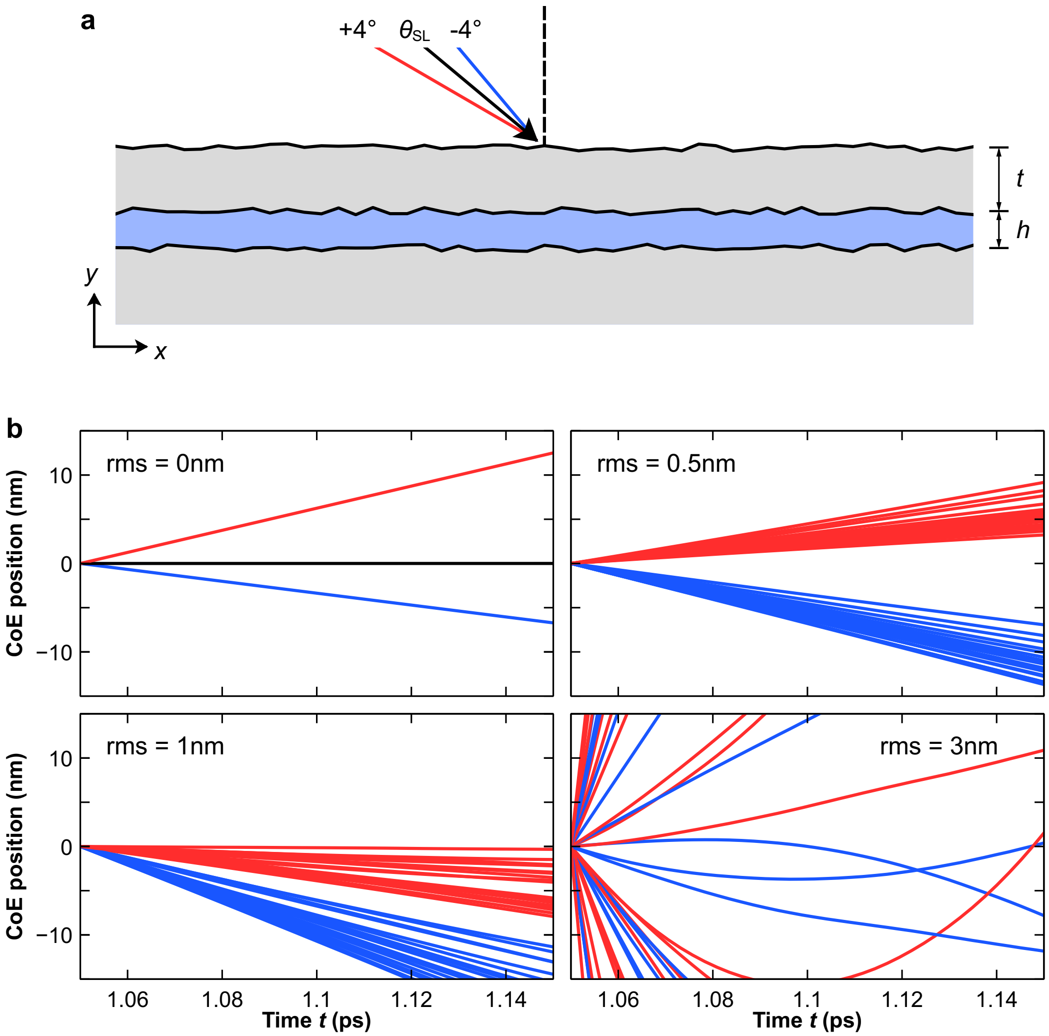

Supplementary Figure 4 | Impact of surface roughness on the existence of SL points in the dispersion relation. (**a**) The slow-light mode is excited by an electromagnetic pulse of Gaussian spatial shape incident under an angle θ and the position of its center of energy (CoE) within the waveguide is subsequently recorded. (**b**) Time-dependence of the CoE position after injection for four values of rms surface roughness. Individual lines correspond to different configurations of the rms surface roughness for incident light under an angle of +4° (blue lines) and -4° (red lines) with respect to the angle $\theta_{\mathrm{SL}}{\sim}17.6^{\circ}$ under which the SL point can be excited in the absence of surface roughness. See "Impact of surface roughness on SL lasing" in the supplementary discussion for further details.

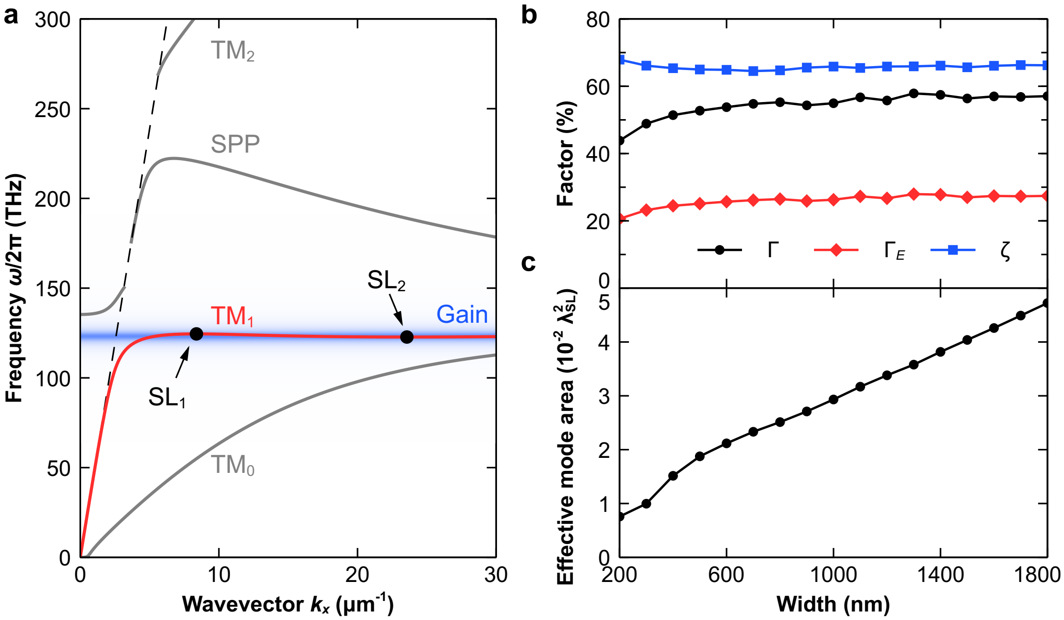

Supplementary Figure 5 | Complex-frequency dispersion and lasing properties of the metal-dielectric stack structure with waveguide core height $\mathbf{h = 110\,nm}$ and top metal layer thickness $\mathbf{t = 50\,nm}$. (**a**) The TM_1 mode with its two SL points, which line up with the gain spectrum (in blue), is highlighted in red. (**b**) Confinement factor Γ and energy confinement Γ_E in dependence of the gain section width during steady-state lasing of the TM_1 mode. The factorization parameter ζ is defined in the text. (**c**) Effective mode area A_{eff} calculated as A_{g}/Γ from the confinement factor in (**b**) and the gain section area $A_{\mathrm{g}} = w \cdot 90\,$nm. See "Steady-state analysis of plasmonic mode" in the supplementary discussion for further details.

Supplementary Tables

Symbol	Description	Value
$\omega_e/2\pi$ (λ_e)	Emission frequency (wavelength)	193.41 THz (1550 nm)
γ_e	Emission spectral half-width	$1/(40\,\mathrm{fs}) = 25\,\mathrm{ps}^{-1}$ ($\sim$32 nm)
$\sigma_{0,e}$	Emission cross-section	$2.09 \cdot 10^{-15}\,\mathrm{cm}^2$
N	Carrier density	$2 \cdot 10^{18}\,\mathrm{cm}^{-3}$
g_e	Maximum gain coefficient	$4180\,\mathrm{cm}^{-1}$

Supplementary Table 1 | Gain medium parameters for emission into the leaky TM_2 waveguide mode.

Symbol	Description	Value
$\omega_a/2\pi$ (λ_a)	Absorption frequency (wavelength)	223.7 THz (1340 nm)
γ_a	Absorption spectral half-width	$1/(40\,\mathrm{fs}) = 25\,\mathrm{ps}^{-1}$ ($\sim$24 nm)
$\sigma_{0,a}$	Absorption cross-section	$2.4 \cdot 10^{-15}\,\mathrm{cm}^2$
g_a	Maximum absorption coefficient	$4810\,\mathrm{cm}^{-1}$

Supplementary Table 2 | Gain medium parameters of the absorption transition.

Symbol	Description	Value
$\omega_e/2\pi$ (λ_e)	Emission frequency (wavelength)	123.89 THz (2419.7 nm)
γ_e	Emission spectral half-width	$1/(63.085\,\mathrm{fs}) \approx 15.85\,\mathrm{ps}^{-1}$ ($\sim$49 nm)
$\sigma_{0,e}$	Emission cross-section	$2.12 \cdot 10^{-15}\,\mathrm{cm}^2$
N	Carrier density	$2 \cdot 10^{18}\,\mathrm{cm}^{-3}$
g_e	Maximum gain coefficient	$4240\,\mathrm{cm}^{-1}$

Supplementary Table 3 | Gain medium parameters for emission into the second stopped light point of the bound TM_1 waveguide mode.

Supplementary Discussion

Optical pumping. In-plane optical pumping of the gain medium is possible at frequencies where the dispersion curve supports propagating modes. Efficient in-coupling of radiation into the waveguide core, for example through a metal grating on top of the thin metal layer or through end-fire coupling, make it possible to optically deliver pump power to the SL lasing region from within the waveguide. In the planar metal-dielectric stack of Figure S2a of the main text, we can use a high-k mode of the TM_2 branch as the pump exploiting the positive curvature of the band's dispersion (see Figure 2b of the main text). For the frequency ω_{pump} of the cw pump field we choose the absorption maximum of the gain medium at $\omega_{\mathrm{a}}/2\pi = 223.7\,\mathrm{THz}$ (see Supplementary Table 2) where $k = 11.31\,\mu\mathrm{m}^{-1}$ and the group velocity is $v_{\mathrm{g}} \approx 0.16c$.

The results for steady-state SL lasing in a setup with 400 nm wide gain section are presented in Supplementary Fig. 1. The pump field enters from the negative x-direction of Supplementary Fig. 1a and, away from the gain section, displays the TM_2 mode profile with its two non-zero components E_x (top) and E_y (bottom). Interference with the lasing mode leads to a strong deviation from the pump mode profile close to and inside the gain section. The interference is particularly strong in the E_x-component, which is the major component of the lasing field. Within this small gain material section of 400 nm width, only about 19% of the pump energy is absorbed.

The field interference observed in Supplementary Fig. 1a can be decomposed into its spectral components by applying a spectral filtering to the steady-state field dynamics at the lasing frequency. Supplementary Figure 1b shows that the resulting mode profile is almost perfectly symmetric around the center of the gain section in propagation direction, which is also the case for the steady-state inversion displayed in Supplementary Fig. 1c. We can conclude that a constant, spatially-homogeneous pump rate r_{p}^{0} will give equivalent results to those presented here as long as only a small proportion of the pump field energy is absorbed within the gain medium.

Density of optical states. The local density of optical states (LDOS) in lossless slow-light photonic systems is enhanced by a factor of $1/v_{\mathrm{g}}$ due to an effective prolongation of the interaction time between the emitter and the fields. This LDOS enhancement leads to a potentially dramatic speed-up of spontaneous emission and can result in strong nonlinear emitter–field interactions through an associated increase in the electric field strength.

Here, we calculate the Purcell factor, i.e. the enhancement of the partial LDOS in

comparison to its free-space value, for a dipole emitter positioned within the waveguide core and polarized to couple either to TM or TE modes of the planar metal-dielectric stack. Subsequent averaging over all positions within the waveguide core yields the average Purcell factor. This enhancement can be compared to[1]

$$F(\omega) = \frac{1}{\rho_0}\frac{1}{h}\int_0^h \mathrm{d}z \int_0^\infty \mathrm{d}k \, \frac{1}{2\pi^2}\frac{\varepsilon_0\varepsilon_\mathrm{s}'\mathbf{E}_k^2(z)}{2\mathcal{E}_k} D(\omega,k) \;, \tag{1}$$

evaluated using the dispersion and field profiles of the waveguide modes. $F_k(\omega)$ is normalized to the free-space value of the partial LDOS, $\rho_0 = \omega/\left(4\pi c^2\right)$ for TM and $\rho_0 = \omega/\left(2\pi c^2\right)$ for TE modes. $D(\omega,k) = \gamma(k)/\left[\pi\left((\omega(k)-\omega)^2+\gamma^2(k)\right)\right]$ is the spectral density of optical states (DOS) and $\mathcal{E}_k = \int \mathrm{d}^3r \, \left[\frac{1}{2}\varepsilon_0 \, \partial\omega\varepsilon'(\mathbf{r},\omega)/\partial\omega|_{\omega(k)} \, \mathbf{E}_k^2(\mathbf{r}) + \frac{1}{2}\mu_0\mathbf{H}_k^2(\mathbf{r})\right]$ is the total energy of the mode at wavevector k. Hence, equation (1) describes an effective weighting of the spectral DOS with the average emitter–field overlap of the mode inside the waveguide core.

In Supplementary Fig. 2, we compare data extracted from FDTD simulations with results obtained from equation (1). We find very good agreement for both the TM and TE modes of the stack waveguide. The Purcell factor for TM modes is about 8 times larger than for TE modes and peaks close to the SL frequencies at 193.8 THz. Due to the positive curvature of the TM_2 modal dispersion the enhancement falls off more slowly towards higher frequencies. The enhanced LDOS directly impacts on the properties of SL lasing, causing an acceleration of the light-matter interaction and a large β-factor as spontaneous emission is predominantly channeled into the SL mode.

Confinement factor and the effective mode volume. Laser rate equations are a set of two coupled differential equations that approximate the dynamics of the photon and carrier number[2]. Applied to nanolasers[3,4] this simple model can reproduce the basic characteristics of a laser, such as its threshold behavior, transient dynamics, and modulation speeds. A comparison between the spatially resolved FDTD simulations and the rate equation model allows us to extract effective parameters of the system. We are particularly interested in the confinement factor and the effective mode volume of SL lasing.

We first note that Poynting's theorem, which describes the evolution of the electromagnetic energy density $U(\mathbf{r},t)$, can be transformed into a rate equation

$$\frac{\partial S}{\partial t} = -\gamma_\mathrm{c} S + R_\mathrm{stim}(N) S \tag{2}$$

for the photon number S at the lasing frequency ω_0 by volume integration of the total energy density, $S = (\hbar\omega_0)^{-1} \int_V d^3r\, U(\mathbf{r}, t)$. This equation also defines the cavity loss rate γ_c and the stimulated emission rate $R_{\mathrm{stim}}(N)$. Fast phase-oscillations of the fields $\mathbf{E}$ and $\mathbf{H}$ at the lasing frequency are eliminated through a time averaging over one period and the integration volume V is taken to encompass the full SL lasing structure making the photon number a slowly-varying, effective variable of the system. In dispersive media, the energy density is given by

$$U(\mathbf{r}, t) = U_E(\mathbf{r}, t) + U_M(\mathbf{r}, t) = \frac{1}{2}\varepsilon_0 \left.\frac{\partial\omega\varepsilon'(\mathbf{r}, \omega)}{\partial\omega}\right|_{\omega=\omega_0} \overline{\mathbf{E}^2(\mathbf{r}, t)} + \frac{1}{2}\mu_0 \overline{\mathbf{H}^2(\mathbf{r}, t)}\ . \qquad (3)$$

Here, $\varepsilon(\mathbf{r}, \omega)$ is the spatially-resolved relative permittivity which follows a Drude dispersion in the metal layers and is equal to ε_a in the active (gain) waveguide core layer (the prime denotes the real part of the complex quantity). It is important to account for the dispersive character of the permittivity to correctly describe the electric energy density U_E, in particular inside metals. In time-domain, this dispersive character is expressed by the dynamic polarization response $\mathbf{P}_f(\mathbf{r}, t)$ of the free electron plasma in the metal and its in-phase $\overline{\dot{\mathbf{P}}_f \cdot \mathbf{E}}$ contribution to the electric energy (see Box 1 in Hess et al.[5]).

In Eq. (2), the cavity loss rate γ_c includes both outcoupling of energy from the laser and dissipation of energy inside the laser. The former is connected to the closed contour integral over the Poynting flux, while the second is given by the work that the fields perform on the free electron plasma of the metal. The rate $R_{\mathrm{stim}}(N)$ arises from stimulated emission of photons in the gain medium with average carrier number N.

When describing semiconductor (microcavity) lasers, and more recently plasmonic nanolasers[3,6,7] using rate analysis, one finds that a confinement factor Γ must be introduced in the rate equations due to the imperfect spatial overlap of the mode profile with the gain section. This confinement factor expresses the fact that the mode volume V_{eff}, which connects the photon density s to the photon number $S = V_{\mathrm{eff}}s$, is distinct from the active (gain) volume V_a, and it is defined as $\Gamma \equiv V_a/V_{\mathrm{eff}}$ [8].

The confinement factor Γ enters the rate of stimulated emission

$$R_{\mathrm{stim}}(N) = \Gamma v_g \zeta g(N)\ , \qquad (4)$$

as does a group velocity v_g (at this point the specific type of group velocity is not fixed), a factorization parameter ζ and the bulk gain coefficient $g(N) = g(\Delta N) = \sigma_a \Delta N$.

The specific definition of the confinement factor and its physical interpretation then determines which group velocity must be used. We follow Chang and Chuang[8] and choose

$$\Gamma \;=\; \frac{\int_{V_\mathrm{a}} d^3r\, \frac{1}{2}\varepsilon_0 \left[\partial(\omega\varepsilon'_\mathrm{a})/\partial\omega + \varepsilon'_\mathrm{a}\right] |\mathbf{E}|^2}{\int_V d^3r\, U} \approx \frac{2\int_{V_\mathrm{a}} d^3r\, U_E}{\int_V d^3r\, U} \;, \tag{5}$$

which identifies v_g as the *material* group velocity of the gain material $v_\mathrm{g} = v_\mathrm{g,a} = c/n_\mathrm{g}$ with $n_\mathrm{g} = \partial(\omega n'_\mathrm{a})/\partial\omega$. The approximation on the rhs of Eq. (5) is valid for a weakly dispersive gain material with $v_\mathrm{g,a} \approx v_\mathrm{ph,a} = c/n'_\mathrm{a}$. The factorization parameter ζ measures the degree of inhomogeneity of the inversion profile (spatial hole-burning) and is defined as

$$\zeta = \frac{\int_{V_\mathrm{a}} d^3r\, \overline{\Delta N(\mathbf{r},t)} \cdot \overline{\mathbf{E}^2(\mathbf{r},t)}}{\Delta N(t) \cdot \int_{V_\mathrm{a}} d^3r\, \overline{\mathbf{E}^2(\mathbf{r},t)}} \;. \tag{6}$$

with the average inversion density $\Delta N(t) = V_\mathrm{a}^{-1} \int_{V_\mathrm{a}} d^3r\, \overline{\Delta N(\mathbf{r},t)}$. Equation (6) expresses a functional dependence of $\zeta(t)$ on the inversion and field intensity profiles and hence an implicit transient dynamics that stabilizes when the laser reaches steady state. In lasers that exhibit negligible spatial hole-burning effects, $\zeta(t)$ is close to unity and can be approximated by a constant factor $\zeta \approx 1$. A time-constant ζ smaller than 1 can also be assumed when the spatial distribution of the inversion and the mode profiles vary only little with time. In these cases, a linear relationship between the stimulated emission rate and the inversion density follows, $R_\mathrm{stim} \propto \Delta N$, a functional dependence that is commonly adopted in rate equation analyses[3].

For further comparison we calculate the stimulated emission rate R_stim from the dynamic change of the total energy, which we are able to extract in FDTD simulations using a rate retrieval method based on Poynting's theorem[5,9]. From Eq. (4) and the knowledge of the confinement factor and average inversion, it is then possible to calculate the factorization parameter ζ. We also compare the confinement factor Γ of the rate equation analysis to the energy confinement $\Gamma_E = \int_{V_\mathrm{a}} d^3r\, U / \int_V d^3r\, U$, which is defined as the electromagnetic energy in the gain section divided by the total energy of the lasing mode. The two confinement factors will differ in strongly guiding or plasmonic systems because of the modal character of the fields, i.e. an unequal distribution of the electromagnetic energy into electric and magnetic components.

As a side note, recent publications on plasmonic nanolasers have suggested the use of a confinement factor defined to incorporate the average energy (or waveguide group)

velocity v_E of the underlying waveguide system of the nanolaser[6,7]. This is possible because the stimulated emission rate R_{stim} and the bulk gain coefficient $g(N)$ in Eq. (4) are invariant to the definition of the effective mode volume, while the confinement factor relates to the group velocity v_{g} used. The distinction into confinement factor and group velocity can therefore be made in terms of waveguide properties, describing the amplification of a wave packet when it travels along the active waveguide with energy velocity v_E. Comparing Γ' in $R_{\mathrm{stim}}(\Delta N) = v_E \Gamma' \zeta g(N)$[6,7] to Γ in Eq. (5), we find the effective confinement factor $\Gamma' = \Gamma v_{\mathrm{g,a}}/v_E$. The pre-factor $v_{\mathrm{g,a}}/v_E$ points towards effective confinement factors Γ' that can become larger than unity in waveguiding systems with low energy velocity. This also implies a divergence of Γ' as $v_E \to 0$, i.e., in the stopped-light regime. Clearly then, Γ' is not suitable for the description of the stationary lasing mode in the SL laser, in particular considering that the stimulated emission rate into the mode, R_{stim}, does not diverge.

Steady-state analysis for varying gain section width. The steady-state properties of the SL laser are here analyzed in dependence of the gain section width. We particularly focus on the confinement factor Γ and the effective mode area A_{eff} of the rate equation analysis as introduced above. The leaky TM_2 mode possesses two SL points at $\omega_1/2\pi = 193.8\,\mathrm{THz}$ ($\lambda_1 \approx 1546.9\,\mathrm{nm}$), $k_1 = 0\,\mu\mathrm{m}^{-1}$ and $\omega_2/2\pi = 193.78\,\mathrm{THz}$ ($\lambda_2 \approx 1547.06\,\mathrm{nm}$), $k_2 = 1.42\,\mu\mathrm{m}^{-1}$. The gain parameters are listed in Supplementary Table 1.

In Supplementary Fig. 3a, we observe that the average inversion ΔN increases sharply for smaller gain section widths w, while the effective modal gain in terms of the stimulated emission rate R_{stim} increases only slightly from $3.66\,\mathrm{ps}^{-1}$ to $3.71\,\mathrm{ps}^{-1}$. These latter values compare very well to mode solver calculations of the total modal loss: $\gamma = 3.62\,\mathrm{ps}^{-1}$ at $k = 0\,\mu\mathrm{m}^{-1}$ increasing to $3.88\,\mathrm{ps}^{-1}$ at $k = 4\,\mu\mathrm{m}^{-1}$. As the localized wave-packet is composed of a range of wavevectors between these limits, the effective loss of the lasing mode calculates as a weighted average of the k-dependent modal losses. Higher localization, as a result of a reduced gain section width, increases the weight of high k components, hence increasing the total loss and with it the stimulated emission rate.

The sharp rise in ΔN for smaller w is linked to an equally sharp reduction in the confinement factor Γ of the lasing mode (Supplementary Fig. 3b); we observe a decrease from above 70% to below 16%. Accordingly, the mode cannot localize fully over the gain section for small widths anymore. Alongside the confinement factor Γ we plot the energy confinement factor Γ_E that has also been extracted numerically in steady state.

The fact that Γ_E does not differ much from Γ gives evidence of the photonic character of the TM_2 mode with its energy being almost equally distributed between electric and magnetic field components. For increasing width w of the gain section, both, Γ and Γ_E asymptotically saturate. The respective values have been determined semi-analytically from mode-solver calculations (plotted as dotted lines in Supplementary Fig. 3b) and are in excellent agreement with those obtained in steady state from the dynamic simulations.

Supplementary Figure 3b also shows that the increase in Γ is to a certain degree mitigated by a decrease in the factorization parameter ζ, which falls from about 90% to just below 50%. The decrease of ζ at large widths is a manifestation of spatial hole burning. As the gain section widens, the SL mode suffers from an increasingly poor overlap with regions at the edge of the gain section where a high inversion builds up. Eventually the spatial hole burning becomes so strong that the SL pulse breaks up and dynamic mode competition sets in. We also note that the TM_2 mode profile has an anti-node in the center of the waveguide core where the inversion can not be depleted. Consequently ζ can not reach a value of 1, even for very small gain section widths.

The effective mode area $A_{\text{eff}} = A_{\text{g}}/\Gamma$ in dependence of the gain section width is displayed in Supplementary Fig. 3c. Despite the strong decrease in Γ the effective mode area A_{eff} shrinks with decreasing w, a characteristic that is accounted for by the linear dependency $A_{\text{g}} \propto w$ and the sub-linear decrease of Γ with w. For small gain section widths A_{eff} eventually levels off. In this regime, where $\Gamma \propto w$, the SL mode retains its field profile and features an almost constant amplitude across the gain section. The smallest measured mode area is obtained for a gain section width of $w = 200\,\text{nm}$ and has a value of $A_{\text{eff}} \approx 0.14\lambda^2$. The results indicate that the subwavelength confinement of the lasing mode in the SL laser is ultimately determined by its dispersion. As the mode is compressed, it becomes (owing to group velocity dispersion) more lossy and, consequently, requires more gain. Once the inversion reaches 100% the losses cannot be compensated by gain anymore and the minimum mode volume is encountered.

Finally, we note that the lasing frequency blue-shifts with decreasing width of the gain section (Supplementary Fig. 3d). This is to be expected because the higher localization of the mode over smaller gain sections is based on the inclusion of increasingly larger wavevector components. The positive curvature of the band accordingly forces the lasing frequency to shift to slightly higher frequencies.

Steady-state analysis of plasmonic mode. A further characterization of the confinement factor and mode area in dependence of the gain section width is shown

in Supplementary Fig. 5. We find a confinement factor Γ that changes only little (from about 57% to 44%) when decreasing w from 1500 down to 200 nm. This is in stark contrast to the properties of the TM_2-based lasing mode for which Γ was seen to decrease from about 70% to 16%. In addition, most of the electromagnetic energy of the TM_1 mode is electric in nature inside the waveguide section, i.e. $\Gamma \approx 2\Gamma_E$. This also leads to a high and fairly constant factorization parameter $\zeta \approx 67\%$, as the mode profile does not feature field nodes along the y-direction. With the confinement factor remaining close to constant, the effective mode area A_{eff} decreases almost linearly with the gain section width (Supplementary Fig. 5b). For this mode, the comparably larger dissipative loss prevents lasing oscillations for small w despite the large final confinement factor of $\Gamma \approx 44\%$ and high $\zeta \approx 68\%$, a result of the plasmonic character of the mode. A mode area of only $A_{\text{eff}} \approx 7.6 \cdot 10^{-3}\lambda^2$ is extracted for the smallest gain section width for which we observe SL lasing.

The superior confinement of the TM_1-based SL lasing mode and its much smaller effective mode volume make this structure an extremely interesting mode for lasing operation in the deep-subwavelength regime at potentially ultra-fast modulation speeds. Energy is emitted from this SL laser in terms of surface plasmons propagating in the plane of the waveguide core layer. This opens the possibility to use the SL laser as a source of surface plasmon polaritons or, equally, to achieve directed emission to free space through grating coupling.

Impact of surface roughness on SL lasing. We analyze a stack structure without gain material where we inject wave packets at two distinct angles corresponding to wavevectors either side of the second SL point. From Supplementary Fig. 4 we find that, up to a critical rms roughness value, the energy velocity remains constant in time with a clear dependence on the injection angle. Hence, below this critical value, one can always find an optimum excitation angle for which the energy velocity along the waveguide direction is zero. It is apparent from the figure that the optimal angle depends on the level of surface roughness and additionally varies from sample to sample. At rms surface roughness of 3 nm, the nature of pulse propagation is changed dramatically as the energy velocity is not constant anymore and the pulse is equally likely to propagate in a forwards or backwards direction. In this regime, the propagation of energy is diffusive and correlates only weakly with the waveguide dispersion. As a result of the strong scattering at the surface inhomogeneities one observes a pulse breakup and the disappearance of the global SL point.

Supplementary References

[1] Iwase, H., Englund, D. & Vučković, J. *Opt. Express* **18**, 16546–16560 (2010).

[2] Björk, G. & Yamamoto, Y. Analysis of semiconductor microcavity lasers using rate equations. *IEEE J. Quant. Electron.* **27**, 2386–2396 (1991).

[3] Oulton, R. F. *et al.* Plasmon lasers at deep subwavelength scale. *Nature* **461**, 629–632 (2009).

[4] Khajavikhan, M. *et al.* Thresholdless nanoscale coaxial lasers. *Nature* **482**, 204–207 (2012).

[5] Hess, O. *et al.* Active nanoplasmonic metamaterials. *Nat. Mater.* **11**, 573–584 (2012).

[6] Ning, C. Z. Semiconductor nanolasers. *Phys. Status Solidi B* **247**, 774–788 (2010).

[7] Ding, K. & Ning, C. Z. Metallic subwavelength-cavity semiconductor nanolasers. *Light: Science & Applications* **1**, e20 (2012).

[8] Chang, S.-W. & Chuang, S. L. Fundamental Formulation for Plasmonic Nanolasers. *IEEE J. Quant. Electron.* **45**, 1014–1023 (2009).

[9] Hamm, J. M., Wuestner, S., Tsakmakidis, K. L. & Hess, O. Theory of light amplification in active fishnet metamaterials. *Phys. Rev. Lett* **107**, 167405 (2011).
